# Atomic Simulations of Grain Structures and Deformation Behaviors in Nanocrystalline CoCrFeNiMn High-Entropy Alloy

**DOI:** 10.3390/ma12071010

**Published:** 2019-03-27

**Authors:** Junling Hou, Qiang Li, Chuanbao Wu, Limei Zheng

**Affiliations:** 1 College of Vanadium and Titanium, Panzhihua University, Panzhihua 617000, China; 13956408171@139.com (J.H.); gswyliqiang@126.com (C.W.); 2 Condensed Matter Science and Technology Institute and Department of Physics, Harbin Institute of Technology, Harbin 150080, China; zhenglm@hit.edu.cn

**Keywords:** molecular dynamics, CoCrFeNiMn, grain boundary, plastic deformation

## Abstract

Using the molecular dynamics method, the melting character, mechanical properties, microstructures, and strain deformation mechanisms of nanocrystalline CoCrFeNiMn high-entropy alloy are systematically investigated in the present work. The simulation results suggest that the melting point in CoCrFeNiMn high-entropy alloy decreases with the grain size, decreasing from 3.6 to 2.0 nm. The grain size has a significant effect on shear and Young’s modulus compared to bulk modulus. The stress-strain simulation demonstrates that the ultimate tensile strength decreases with the decrease of the grain size, while the plastic deformation increases with the decrease in grain size. While the average grain size decreases to 2.0 nm, the amorphization induced by small grain size reduces plastic deformation. The common neighbor analysis shows that the face-centered cubic (FCC) composition of CoCrFeNiMn decreases gradually with decreasing grain size. For the sample with a grain size of 2.0 nm, the FCC composition is about 19% at a strain of 20%, accompanied by severe amorphization. The inverse Hall-Petch effect is observed for nanocrystalline CoCrFeNiMn high-entropy alloy in the present simulations. The atomic snapshot of CoCrFeNiMn with a grain size of 2.0 nm under the uniaxial strain confirms that the grain shape change, stacking fault formation, and amorphization are important mechanisms of plastic deformation in nanocrystalline high-entropy CoCrFeNiMn.

## 1. Introduction

There are many well-known advantages to using high entropy alloys (HEAs), e.g., exceptional high-temperature strength, wear resistance, and excellent low-temperature ductility [1,2,3], thus researchers pay more attention to developing new HEAs. CoCrFeNiMn is an appealing high-entropy alloy, exhibiting high strength and high ductility. In recent decades, the researchers have investigated CoCrFeNiMn high-entropy alloy using experimental methods. Salishchev et al. [4] studied the microstructure and mechanical properties of the equiatomic composition alloys FeCrCoNi, FeCrCoNiV, FeCrCoNiMn, and FeCrCoNiMnV under as-solidified and annealed conditions. They found that the FeCrCoNi and FeCrCoNiMn alloys are single-phase face-centered cubic (FCC) structures in both conditions, and that the CoCrFeNi and CoCrFeNiMn alloys are soft and extremely ductile whereas the CoCrFeNiV and CoCrFeNiMnV alloys are hard and strong. Using equal-channel angular pressing, Shahmir et al. [5] investigated the mechanical properties of CoCrFeNiMn high-entropy alloy and found that the strength increases gradually with the increase of strain to 1 GPa with an elongation to failure of about 35% after four passes. The microstructure is a single phase with an average grain size of about 100 nm, and the grain boundary strengthening is the most important strengthening mechanism in CoCrFeNiMn. Stepanov et al. [6] studied the effect of thermomechanical processing on the microstructure of the carbon-containing CoCrFeNiMn high-entropy alloy, and their results showed that the addition of carbon increases dislocation activity while simultaneously retarding deformation twinning during rolling and decreases the fraction of twin boundaries in the annealed condition. Heczel et al. [7] used the high-pressure torsion (HPT) method to investigate the defect structure and hardness in nanocrystalline CoCrFeMnNi high-entropy alloy, and found that the grain size was gradually refined from ~60 μm to ~30 nm while the dislocation density and the twin-fault probability increased to very high values. After two series of HPT, the microhardness increased from ~1440 MPa to ~5380 MPa. Yu et al. [8] prepared CoCrFeCuNi and CoCrFeMnNi high-entropy alloys via mechanical alloying and high-pressure sintering (HPS) and found that the bulk modulus is 117.5 GPa and 136.1 GPa for the HPSed CoCrFeCuNi and CoCrFeMnNi HEAs, respectively. Dang et al. [9] adapted high-vacuum radio frequency magnetron sputtering to obtain an equiatomic CoCrFeMnNi high-entropy alloy thin film and investigated its mechanical properties. Their results showed that the deposition of a smooth and homogenous thin film with a grain size of ~10 nm is achieved through this technique. This thin film has a high hardness of 6.8 ± 0.6 GPa, which is superior compared to its bulk counterpart owing to its nanocrystalline structure. Theoretically, Tian et al. [10] studied the magnetic states of CoCrFeMnNi high-entropy alloy, and they proposed that the paramagnetic state increases the mechanical stability of the FCC phase against tetragonal deformation as compared to the ferromagnetic state. Based on molecular dynamics computer simulations, Korchuganov [11] researched the mechanical response of bulk Co_10_Cr_10_Fe_30_Mn_30_Ni_20_ and Co_30_Cr_30_Fe_10_Mn_10_Ni_20_ and found that these two stoichiometric compositions have Young’s moduli of 81 GPa and 103 GPa, respectively, and the plasticity in the single crystal CoCrFeMnNi nucleates through the formation of intrinsic stacking faults during high-rate compression or tension.

Despite the experimental and theoretical studies carried out so far, the mechanical response in nanocrystalline CoCrFeNiMn high-entropy alloy has not yet been reported in the literature. Thus, this prompted us to study the microstructure structure and mechanical properties for CoCrFeNiMn high-entropy alloy with nanometric diameter grain sizes, which would reinforce the knowledge of CoCrFeNiMn high-entropy alloy. The molecular dynamics method can predict various properties of materials, so it has been selected for this purpose.

## 2. Materials and Methods

Molecular dynamics (MD) simulations were performed using the LAMMPS software (Sandia National Laboratories, Philadelphia, PA, USA) [12], based on Newtonian motion equations. The interatomic interactions were described by the modified embedded-atom method potential [13]. The integration step for the MD calculations was set to 2 fs. All models were completely equilibrated for 30,000 time steps under periodic boundary conditions in a Nose-Hoover NPT ensemble at 0 GPa and 300 K, and then the systems were melted from 300 K up to 3000 K during 9000 time steps in order to simulate the melting processes. For the tensile testing simulation, the strain rate along the *x* direction was set to 0.05%/ps, while the NPT ensemble was applied in the *y* and *z* directions with the periodic boundary condition. The four seed models of CoCrFeNiMn high-entropy alloy with an average grain size of 3.6–2.0 nm were built based on Voronoi construction through the Atomsk [14]. The OVITO software [15] was selected for visualization of the atomic snapshots of the seed samples. Identification of the microstructure of the samples was conducted on the basis of an algorithm determining the symmetry of the nearest-neighbor atom, i.e., common neighbor analysis (CNA). According to the CNA, an intrinsic stacking fault (SF) in the FCC lattice was defined as two layers of atoms with HCP symmetry of the nearest-neighbor environment. To calculate the elastic constants, the stress-strain method in the LAMMPS software was used in the present work. Firstly, the models were relaxed for 30,000 time steps in Nose-Hoover NPT ensemble at 0 GPa and 300 K. Secondly, an NVT simulation of 2000 time steps was run to obtain the average stress tensor under the six different cell deformations of 0.05. Finally, the elastic constants of the models were obtained through Hooke’s law.

## 3. Results

### 3.1. Melting Properties

**Figure 1:**
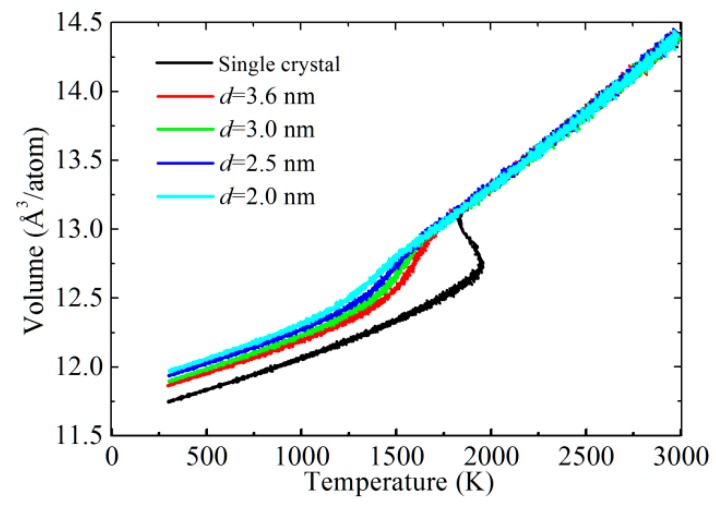
Volume as a function of temperature for CoCrFeNiMn high-entropy alloy with different grain sizes.

Using the present interatomic potential, the calculated lattice parameter of CoCrFeNiMn high-entropy alloy with a perfect FCC structure is about 0.3608 nm at 300 K and 0 GPa, which is agreement with the experimental values (0.3590 nm [16] and 0.3592 nm [17]). In order to obtain valuable information on the melting properties of CoCrFeNiMn high-entropy alloy, we calculated the volume-temperature curve, as shown in Figure 1. We found that CoCrFeNiMn high-entropy alloy with a perfect FCC structure shows a large change in volume at 1940 K, which indicates that the melting point of a single crystal of CoCrFeNiMn is located in this region. When the average grain size is 3.6 nm, the volume change of CoCrFeNiMn with the increase of temperature is not drastic, and the melting point occurs at around 1430 K. While the grain size decreases from 3.6 nm to 2.5 nm, the corresponding melting point decreases to 1340 K, which suggests that the melting point in CoCrFeNiMn high-entropy alloy decreases with the decrease of the grain size. For the seed model with an average grain size of 2.0 nm, the large volume change in the temperature range of 300–3000 K would be difficult to observe because of amorphization caused by small grain size. This will be discussed later in more detail later. The volume-temperature curves of all models are the same above 2000 K, implying that the molten state of all samples is only the function of temperature but has no relation to the grain sizes.

We also calculate the diffusion coefficient of CoCrFeNiMn through the following equation [18]:

(1) $$D=\underset{t\to \infty }{\lim}\frac{1}{6Nt}\langle \sum_{i=1}^{N}|r_{i}\left(t\right)-r_{i}\left(0\right){|}^{2}\rangle ,$$

where *N* is the number of particles, $r_{i}\left(t\right)$ and $r_{i}\left(0\right)$ are the initial position and final position of the particle *i* after the time *t*. The predicted average diffusion coefficient of CoCrFeNiMn is about 15.0 × 10^−9^ m^2^·s^−1^ at the temperature of 3000 K, and the diffusion coefficient of Mn is highest among all elements, as shown in Table 1.

### 3.2. Elastic Properties

**Figure 2:**
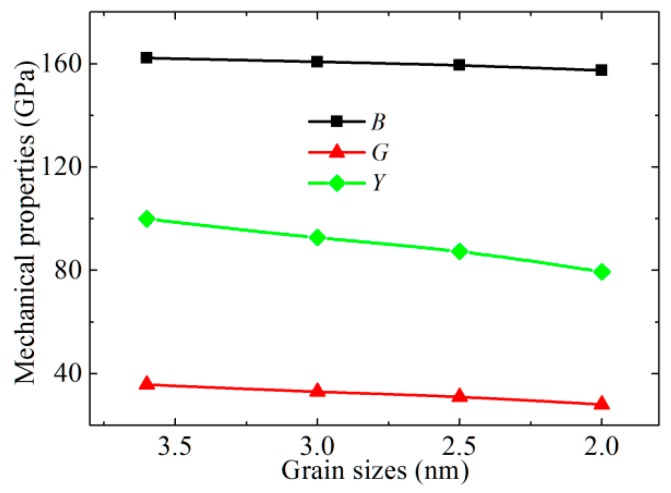
The bulk, shear, and Young’s modulus properties of CoCrFeNiMn with different mean grain sizes.

Through the stress–strain method, the mechanical properties of CoCrFeNiMn with the different grain sizes are calculated, and the results are shown in Figure 2. We presume that the four seed models have cubic symmetry. For the cubic structure, there are three independent elastic constants, i.e., *C*_11_, *C*_12_, and *C*_44_. From the calculated elastic constants *C_ij_*, some mechanical parameters of CoCrFeNiMn, e.g., bulk modulus (*B*) and shear modulus (*G*), can be calculated through the Voigt approximation [19]. The Voigt *B* can be given by:

(2) $$B=\frac{1}{9}(c_{11}+c_{22}+c_{33})+\frac{2}{9}(c_{12}+c_{13}+c_{23}).$$

The Voigt *G* is defined as:

(3) $$G=\frac{1}{15}(c_{11}+c_{22}+c_{33}-c_{12}-c_{13}-c_{23})+\frac{1}{5}(c_{44}+c_{55}+c_{66}).$$

It is found from Figure 2 that with the increase of grain size, the bulk modulus of CoCrFeNiMn decreases slightly, indicating that the effect of grain size on the bulk modulus is quite weak. The case of shear modulus is different from that of bulk modulus, and the corresponding decreasing trend is significant. As the grain size decreases from 3.6 to 2.0 nm, the shear modulus of CoCrFeNiMn is reduced by 22%. The case of Young’s modulus (*Y*) is similar to that of shear modulus, while its reducing magnitude is about 20%. We also calculate the *B*, *G*, and *Y* of single crystal CoCrFeNiMn through the equations above, and the corresponding quantities are 169, 56, and 151 GPa, respectively. Because the CoCrFeNiMn alloy has a complex composition and the mechanical properties would be affected by the experiment condition, there exists some deviations between our theoretical and the experimental values [20,21].

### 3.3. Stress–Strain Relation

**Figure 3:**
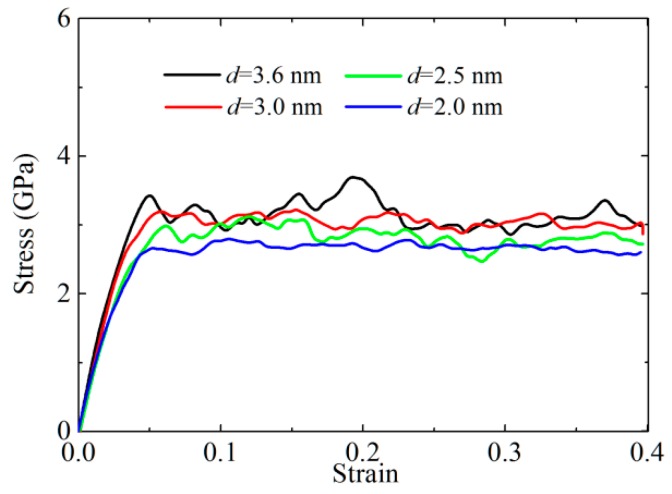
The stress-strain curve of CoCrFeNiMn with different mean grain sizes at 300 K.

The stress–strain curves of CoCrFeNiMn at the temperature of 300 K and for the mean grain sizes of 3.69–2.0 nm under uniaxial tensile strain are depicted in Figure 3. Firstly, the stress–strain curves increase linearly below a strain of 2%, which is corresponding to the elastic region. Then, the increasing trend becomes gentler above 2%, indicating that the elastic-to-plastic transition occurs in this region. With a further increase in strain, the stress–strain curves continuously increase, and the stresses reach the ultimate tensile strength when the strain is about 5–6%. In the region of 7–40%, plastic flow is obtained. For the models with mean grain sizes of 3.6, 3.0, and 2.5 nm, we find that their ultimate tensile strengths (i.e., 3.4, 3.2, and 3.0 GPa, respectively) occur at a strain of 5.1, 5.7, and 6.3%, respectively, which suggests that the strength decreases with the decrease of mean grain size, while the plastic deformation increases with the decrease in grain size. It is noted that the ultimate tensile strength of CoCrFeNiMn with the average grain size of 2.0 nm is located at a strain of 5%, which is ascribed to the amorphization induced by small grain size.

The flow stresses are given by the average values of stresses in the strain range of 10–40%. By fitting both of the flow stresses and grain size to a linear equation, the inverse Hall–Petch relation can be obtained by

(4) $$\sigma_{F}=2.16286+0.28096\cdot d,$$

where *d* is grain size. From Equation (4), we can see that the flow stress increases gradually with grain size.

### 3.4. Structure Character under Uniaxial Strain

**Figure 4:**
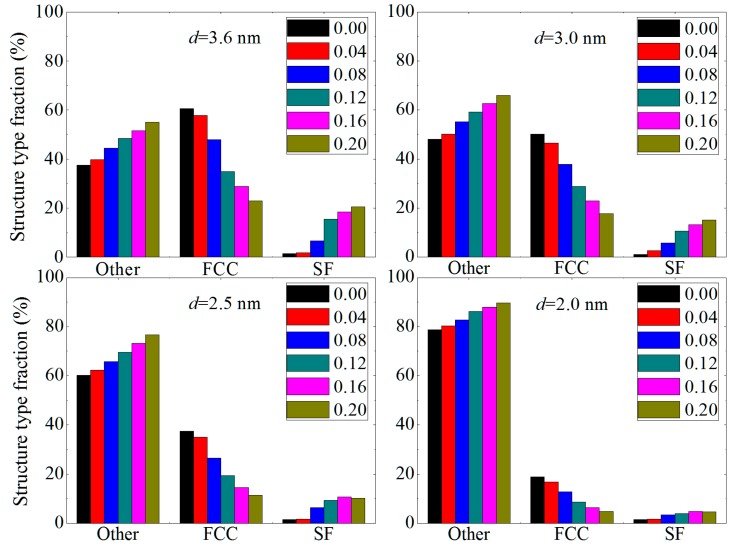
The structure type fraction of CoCrFeNiMn with different mean grain sizes under uniaxial tension strain. The FCC, SF and other represents the face-centered cubic, stacking fault and amorphous structure, respectively.

In order to obtain information on structure change under uniaxial tension strain, we apply the CNA method to plot the structure type fraction of CoCrFeNiMn with different average grain sizes, as shown in Figure 4.

It is found that while the strain is 0.0, the FCC composition of CoCrFeNiMn with *d* = 3.6 nm is about 60%. When *d* decreases to 3.0 nm, the FCC composition of CoCrFeNiMn becomes 50%. With *d* decreasing to 2.5 nm, the FCC composition decreases to about 38%. This indicates that the FCC composition of CoCrFeNiMn decreases gradually with decreasing grain size. For the CoCrFeNiMn alloy with *d* = 2.0 nm, the FCC composition is only 19%, which suggests that the CoCrFeNiMn alloy shows a severe amorphous phenomenon because the grain size becomes small enough. With increasing strain, the other structure (i.e., the amorphous phase) of CoCrFeNiMn with *d* = 3.6–2.0 nm increases gradually, which suggests that the strain can lead to amorphization. This is in agreement with the decreasing FCC composition. Furthermore, the stacking fault of CoCrFeNiMn with *d* = 3.6–2.0 nm also increases with the decrease of the grain size, which suggests that under uniaxial tension strain, the stacking fault contributes to the plastic deformation of CoCrFeNiMn. This contribution would decrease gradually with decreasing grain size.

### 3.5. Deformation Mechanism under Uniaxial Tension

**Figure 5:**
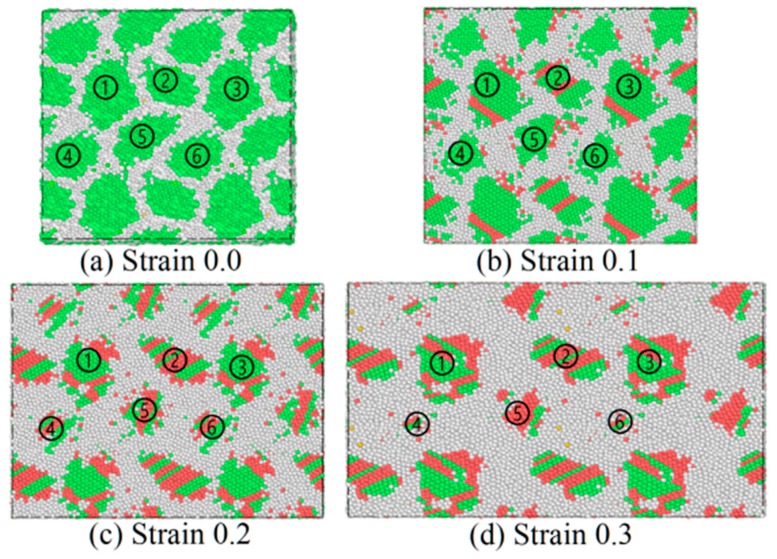
Atom snapshot of CoCrFeNiMn high-entropy alloy with the seed grain size of 3.0 nm stretched at a strain rate of 5 × 10^8^/s and a temperature of 300 K colored by CNA values. (**a**) Strain 0.0; (**b**) Strain 0.1; (**c**) Strain 0.2; (**d**) Strain 0.3.

On a macro scale, the mechanism of plastic deformation for the polycrystalline alloy contributes to the dislocation nucleation and motion. However, when the grain size is decreased to the nanometer scale, the grain-boundary mediated process would be the main deformation characteristic. In this section, the detailed atomic snapshot of CoCrFeNiMn high-entropy alloy at a strain rate of 5 × 10^8^/s is investigated. The atomic snapshots of CoCrFeNiMn with the grain size of 3.0 nm colored by the CNA values are presented in Figure 5. Figure 5a displays the relaxed structure after equilibration at 300 K, where we digitally identify six grains. It is found that the equilibration structure has complete grain boundaries. At a strain of 10%, the thickness of grain boundaries between the grain 4 and 5 (or the grain 5 and 6) increase, while some stacking fault occurs in the grain 1, 2 and 3. When the strain increases to 20%, the thickness of grain boundaries between the grains 4 and 5 (or grains 5 and 6) become thicker than before, accompanied by the decreasing grain size and the appearance of stacking faults. Grains 1–3 incur a change in shape, and the stacking fault composition in grains 1–3 increases. When the strain is up to 30%, grains 4 and 6 almost disappear, and the shape of grains 1–3 is very different from that at a strain of 10%. The amorphous phase caused by the strain is larger than the FCC phase. Therefore, the grain shape change, stacking fault formation, and amorphization are the main mechanisms of plastic deformation for CoCrFeNiMn high-entropy alloy.

## 4. Conclusions

The melting behavior, mechanical properties, and deformation mechanisms of nanocrystalline CoCrFeNiMn high-entropy alloy have been investigated using molecular dynamics simulation. The results suggest that the melting point of CoCrFeNiMn high-entropy alloy increases with the increase of grain size, and the diffusion coefficient of Mn in the molten CoCrFeNiMn is highest among all elements. The elastic calculations show that the effect of grain size on bulk modulus is very slight. The case of shear modulus is different from that of bulk modulus. As the grain size decreases from 3.6 nm to 2.0 nm, the shear modulus and Young’s modulus are reduced by 22% and 20%, respectively. The stress–strain curves demonstrate that the ultimate tensile strength decreases with a decrease in mean grain size, while the plastic deformation increases with a decrease in average grain size. When the mean grain size decreases to 2.0 nm, the amorphization induced by small grain size causes the plastic deformation to decrease. The relationship between the flow stresses and grain size meets the inverse Hall–Petch relation. The CNA analysis shows that the FCC composition of CoCrFeNiMn decreases gradually with decreasing grain size. For the samples with *d* = 2.0 nm, the FCC composition is only 19%, accompanied by a severe amorphous phenomenon because of the small grain size. An atomic snapshot of CoCrFeNiMn with *d* = 2.0 nm under the uniaxial strain analysis confirms that the grain shape change, stacking fault formation, and amorphization are the main mechanisms of plastic deformation for nanocrystalline CoCrFeNiMn high-entropy alloy.

## Figures and Tables

**Table 1:** The calculated diffusion coefficient for CoCrFeNiMn at 3000 K.

Atom Type	Co	Cr	Fe	Ni	Mn	CoCrFeNiMn
Diffusion coefficient (m^2^·s^−1^)	15.3 × 10^−9^	14.7 × 10^−9^	14.5 × 10^−9^	14.3 × 10^−9^	16.5 × 10^−9^	15.0 × 10^−9^

## References

[1] Zhang W., Liaw P.K., Zhang Y. (2018). Science and technology in high-entropy alloys. Sci. China Mater..

[2] Miracle D.B., Senkov O.N. (2017). A critical review of high entropy alloys and related concepts. Acta Mater..

[3] Zhang Y., Zuo T.T., Tang Z., Gao M.C., Dahmen K.A., Liaw P.K., Lu Z.P. (2014). Microstructures and properties of high-entropy alloys. Prog. Mater. Sci..

[4] Salishchev G.A., Tikhonovsky M.A., Shaysultanov D.G., Stepanov N.D., Kuznetsov A.V., Kolodiy I.V., Tortika A.S., Senkov O.N. (2014). Effect of Mn and V on structure and mechanical properties of high-entropy alloys based on CoCrFeNi system. J. Alloy. Compd..

[5] Shahmir H., Mousavi T., He J.Y., Lu Z.P., Kawasaki M., Langdon T.G. (2017). Microstructure and properties of a CoCrFeNiMn high-entropy alloy processed by equal-channel angular pressing. Mater. Sci. Eng. A.

[6] Stepanov N.D., Shaysultanov D.G., Chernichenko R.S., Yurchenko N.Y., Zherebtsov S.V., Tikhonovsky M.A., Salishchev G.A. (2017). Effect of thermomechanical processing on microstructure and mechanical properties of the carbon-containing CoCrFeNiMn high entropy alloy. J. Alloy. Compd..

[7] Heczel A., Kawasaki M., Labar J.L., Jang J.I., Langdon T.G., Gubicza J. (2017). Defect structure and hardness in nanocrystalline CoCrFeMnNi High-Entropy Alloy processed by High-Pressure Torsion. J. Alloy. Compd..

[8] Yu P.F., Zhang L.J., Cheng H., Zhang H., Ma M.Z., Li Y.C., Li G., Liaw P.K., Liu R.P. (2016). The high-entropy alloys with high hardness and soft magnetic property prepared by mechanical alloying and high-pressure sintering. Intermetallics.

[9] Dang C.Q., Surjadi J.U., Gao L.B., Lu Y. (2018). Mechanical Properties of Nanostructured CoCrFeNiMn High-Entropy Alloy (HEA) Coating. Front. Mater..

[10] Tian F.Y., Varga L.K., Shen J., Vitos L. (2016). Calculating elastic constants in high-entropy alloys using the coherent potential approximation: Current issues and errors. Comp. Mater. Sci..

[11] Korchuganov A.V. (2018). Onset of plastic deformation in non-equiatomic fcc CoCrFeMnNi high-entropy alloys under high-rate loading. Lett. Mater..

[12] Plimpton S. (1995). Fast Parallel Algorithms for Short-Range Molecular Dynamics. J. Comput. Phys..

[13] Choi W.-M., Jo Y.H., Sohn S.S., Lee S., Lee B.-J. (2018). Understanding the physical metallurgy of the CoCrFeMnNi high-entropy alloy: an atomistic simulation study. npj Comput. Mater..

[14] Hirel P. (2015). Atomsk: A tool for manipulating and converting atomic data files. Comput. Phys. Commun..

[15] Stukowski A. (2009). Visualization and analysis of atomistic simulation data with OVITO–the Open Visualization Tool. Model. Simul. Mater. Sci. Eng..

[16] Bhattacharjee P.P., Sathiaraj G.D., Zaid M., Gatti J.R., Lee C., Tsai C.-W., Yeh J.-W. (2014). Microstructure and texture evolution during annealing of equiatomic CoCrFeMnNi high-entropy alloy. J. Alloy. Compd..

[17] Stepanov N.D., Shaysultanov D.G., Salishchev G.A., Tikhonovsky M.A., Oleynik E.E., Tortika A.S., Senkov O.N. (2015). Effect of V content on microstructure and mechanical properties of the CoCrFeMnNiV_x_ high entropy alloys. J. Alloy. Compd..

[18] Yan H.-J., Zhuang J.-C., Zhou P., Li Q., Zhou C.Q., Fu P. (2017). Molecular dynamics simulation of thermal physical properties of molten iron. Int. J. Heat Mass. Tran..

[19] Hill R. (1963). Elastic properties of reinforced solids: Some theoretical principles. J. Mech. Phys. Solids..

[20] Alagarsamy K., Fortier A., Mishra R., Kumar N. Investigation of Thermo-Mechanical Processing and Mechanical Properties of CoCrFeNiMn High Entropy Alloy for Peripheral Vascular Stent Application. Proceedings of the ASME 2016 11th International Manufacturing Science and Engineering Conference.

[21] Laplanche G., Gadaud P., Horst O., Otto F., Eggeler G., George E.P. (2015). Temperature dependencies of the elastic moduli and thermal expansion coefficient of an equiatomic, single-phase CoCrFeMnNi high-entropy alloy. J. Alloy. Compd..

